# Characterization of Extra-Cellular Vesicle Dielectrophoresis and Estimation of Its Electric Properties

**DOI:** 10.3390/s22093279

**Published:** 2022-04-25

**Authors:** Hao Chen, Tsubasa Yamakawa, Masafumi Inaba, Michihiko Nakano, Junya Suehiro

**Affiliations:** 1 Graduate School of Information Science and Electrical Engineering, Kyushu University, 744 Motooka, Nishi-ku, Fukuoka 819-0395, Japan; chen@hv.ees.kyushu-u.ac.jp; 2 Department of Engineering, Kyushu University, 744 Motooka, Nishi-ku, Fukuoka 819-0395, Japan; yamakawa@hv.ees.kyushu-u.ac.jp; 3 Faculty of Information Science and Electrical Engineering, Kyushu University, 744 Motooka, Nishi-ku, Fukuoka 819-0395, Japan; inaba@ees.kyushu-u.ac.jp (M.I.); suehiro@ees.kyushu-u.ac.jp (J.S.)

**Keywords:** exosome, liquid biopsy, membrane capacitance, inner conductivity, cancer

## Abstract

Dielectrophoresis (DEP) refers to a type of electrical motion of dielectric particles. Because DEP is caused by particle polarization, it has been utilized to characterize particles. This study investigated the DEP of three types of exosomes, namely bovine milk, human breast milk, and human breast cancer exosomes. Exosomes are kinds of extracellular vesicles. The crossover frequencies of the exosomes were determined by direct observation of their DEPs. Consequently, bovine and human milk exosomes showed similar DEP properties, whereas the cancer exosomes were significantly different from the others. The membrane capacitance and conductivity of the exosomes were estimated using determined values. A significant difference was observed between bovine and human milk exosomes on their membrane capacitance. It was revealed that the membrane capacitances of human breast milk and human breast cancer exosomes were almost identical to those of their host cells and the conductivity of the exosomes were much lower than that of the host cell. Based on these results, DEP separation of the human breast milk and cancer exosomes was demonstrated. These results imply that DEP can be utilized to separate and identify cancer exosomes rapidly. Additionally, our method can be utilized to estimate the electric property of other types of extracellular vesicles.

## 1. Introduction

Dielectrophoresis (DEP) is the electrokinetic motion of a small dielectric particle subjected to a non-uniform electric field. Many DEP applications for manipulating biological samples, such as bacteria, viruses, liposomes, proteins, and DNA suspended in aqueous solutions, have been demonstrated, especially in a microscopic space, such as a microchannel [1,2]. The magnitude and direction of the DEP force depend on the polarization of the dielectric particles. Since the groundbreaking research on the utilization of DEP in the separation of live and dead cells [3], studies related to the DEP-based characterization of biological samples have been carried out [4,5,6,7,8,9,10]. Polarization depends on the conductivity and permittivity of the cell components, such as the cell membrane and cytoplasm. Therefore, such properties of the target can be determined by observing the DEP phenomena.

Recently, among extracellular vesicles, exosomes 30–200 nm in diameter have received attention because there is a possibility that their analysis can be used for the liquid biopsy of cancer or other diseases [11,12,13]. Exosomes are released endosomes; they contain proteins, DNA, and RNA derived from the host cells. It has been found that the characteristics of the exosome membrane, including membrane proteins and carbohydrate sugar chains, are similar to those of the host cell.

Exosomes can be found in several physiological samples such as blood, urine, milk, and saliva. To analyze exosomes, it is necessary to separate them from the samples. Typically, ultracentrifugation and size-exclusion chromatography have been used. Separation using DEP has also been studied [6,10,14,15,16]. Exosomes may experience positive DEP in the typical physiological condition because of their small size. In contrast, negative DEP occurs on larger biological samples, such as cells.

DEP can be utilized not only for separation but also for characterization. To the best of our knowledge, no study has characterized exosomes by directly observing the DEP phenomena. One related study can be found in which DEP affected the streaming of fluorescently-labeled exosomes in a microfluidic device [17]. From the DEP phenomena, the electric properties of exosomes can be estimated. This estimation may reveal the electrical characterization of the specific exosome, such as that of a cancer cell. Some studies have found specific DEP properties in cancer cells [6,10,18,19,20]. Their DEP properties were different from those of normal cells because of the structure and components of the cell membrane. These studies showed the possibility of determining cancer cells by analyzing their DEP properties. Moreover, the investigation of their DEP properties enables the separation of cancer cells from normal cells using the specific frequency of DEP which forces positive and negative DEP on cancer and normal cells, respectively.

As mentioned earlier, the components and exosome membranes are derived from the host cell [11,12,13]. The characteristics of exosomes provide important information for the liquid biopsy of cancers. DEP experiments are simple, rapid, and cost less to analyze a target as compared to typical molecular biology techniques such as electrophoresis, centrifugation, and sequencing. The observed exosome characteristics help separate and diagnose exosomes from cancer cells.

In this study, the DEP properties of three types of exosomes derived from bovine milk, human breast milk, and human breast cancer were investigated by direct observation. The electrical properties of each component were estimated. The crossover frequencies with a series of suspension medium conductivities were determined as the DEP property. It was found that the DEP properties of bovine and human milk exosomes were similar. Exosomes from human breast cancer cells were significantly different from the others. The exosome membrane capacitance and the conductivity of the inside exosome were estimated.

## 2. Theory

### DEP of Exosome: Single-Shell Model

DEP is the electrokinetic motion of a dielectric particle subjected to a non-uniform electric field. This phenomenon is caused by the polarization of the particle [1,4]. The DEP force ($F_{\mathrm{DEP}}$) acting on a spherical particle is given by the following equation:

(1) FDEP=2πr3εmRe[K(ω)]∇|E|2

where r is the radius of the particle, $\varepsilon_{m}$ is the permittivity of the surrounding medium, and *E* is the magnitude of the applied electric field. In this study, an exosome is considered as a sphere as TEM observation by other literature [21,22,23]. Re[K(ω)] is the real component of the Clausius–Mossotti (CM) factor and K(ω) is given by the following equation:

(2) $$K\left(\omega \right)=\frac{\varepsilon_{p}{}^{*}-\varepsilon_{m}{}^{*}}{\varepsilon_{p}{}^{*}+2\varepsilon_{m}{}^{*}}$$

where $\varepsilon_{p}^{*}$ and $\varepsilon_{m}^{*}$ are the complex permittivities of the particle and the surrounding medium, respectively. The complex permittivity is given by the following equation:

(3) $$\varepsilon^{*}=\varepsilon -j\frac{\sigma }{\omega }$$

where σ and ω are the conductivity and angular frequency, respectively.

The CM factor corresponds to the polarizability of the particles. If the dielectric particle polarization is higher than that of the surrounding medium, Re[K(ω)]>0, the particle moves towards the strong electric field region. This phenomenon is referred to as positive DEP (p-DEP). In contrast, with low particle polarization, Re[K(ω)]<0, the particle moves to the weak electric field region, and is called negative DEP (n-DEP). When a thin microelectrode is used for DEP application, the particles are trapped on and repelled from the microelectrode by p-DEP and n-DEP, respectively. The DEP force does not occur if there is no difference in polarization between the particle and the surrounding medium.

An exosome can be considered as the single-shell model. A spherical particle can be considered a homogeneous inner core covered with a thin membrane in the model. The complex permittivity of the single-shell model can be expressed as follows:

(4) $$\varepsilon_{\mathrm{exosome}}^{*}=\varepsilon_{\mathrm{mem}}^{*}\frac{{\left(\frac{r_{i}+r_{\mathrm{mem}}}{r_{i}}\right)}^{3}+2\left(\frac{\varepsilon_{i}^{*}-\varepsilon_{\mathrm{mem}}^{*}}{\varepsilon_{i}^{*}+2\varepsilon_{\mathrm{mem}}^{*}}\right)}{{\left(\frac{r_{i}+r_{\mathrm{mem}}}{r_{i}}\right)}^{3}-\left(\frac{\varepsilon_{i}^{*}-\varepsilon_{\mathrm{mem}}^{*}}{\varepsilon_{i}^{*}+2\varepsilon_{\mathrm{mem}}^{*}}\right)}$$

where $r_{i}$ and $\varepsilon_{i}^{*}$ are the radius and complex permittivity of the inner core, respectively, and $r_{\mathrm{mem}}$ and $\varepsilon_{\mathrm{mem}}^{*}$ are the thickness and complex permittivity of the thin membrane, respectively.

By using Equations (1)–(4) to fit the results of the observed DEP phenomena, the electrical parameters can be estimated. However, it is difficult to determine the magnitude of the DEP force because the DEP force depends on the particle position, $\nabla {\left|E\right|}^{2}$. Therefore, the crossover frequency of the DEP was determined to characterize the target particles [5,7,8,10,20,24]. At the crossover frequency, the DEP force becomes zero. The frequency showing no DEP force can be determined by direct observation with the application of a frequency sweep voltage.

**Figure 1:**
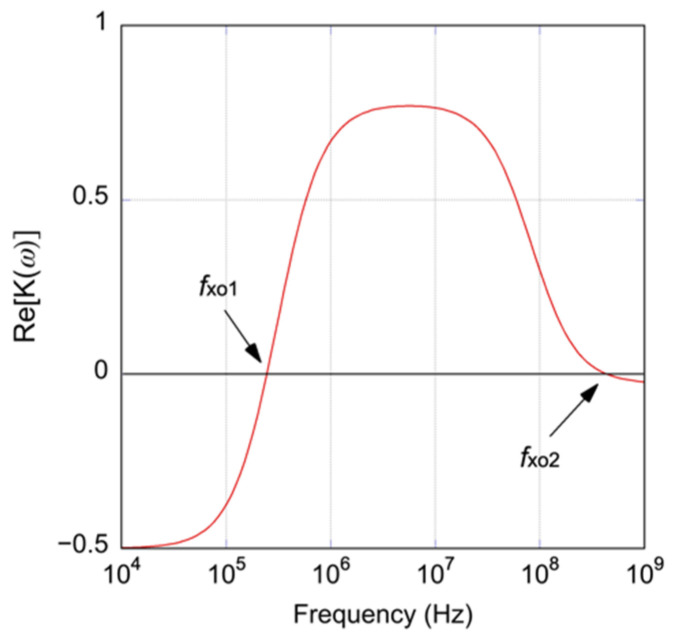
Typical frequency response of Re[K(ω)]. $f_{\mathrm{xo}1}$ and $f_{\mathrm{xo}2}$ are the crossover frequencies.

There are two crossover frequencies in the single-shell model, as shown in Figure 1. In this study, the lower crossover frequency, $f_{\mathrm{xo}1}$, was investigated. The crossover frequency, $f_{\mathrm{xo}1}$, can be written as follows [25,26]:

(5) $$f_{\mathrm{xo}1}=\frac{1}{\sqrt{2}\pi RC_{\mathrm{mem}}}\left(\sigma_{m}-\frac{1}{2\sigma_{i}}\sigma_{m}{}^{2}\right)+f_{\mathrm{xo}}$$

where $f_{\mathrm{xo}1}$ is the extrapolated value to the crossover frequency at $\sigma_{m}=0$, $f_{\mathrm{xo}1}$ was treated as a fitting parameter, and $C_{\mathrm{mem}}$ is the capacitance of the membrane $R=r_{i}+r_{\mathrm{mem}}$. In the experiment, $f_{\mathrm{xo}1}$ at various $\sigma_{m}$ values were determined. Then, the values of $C_{\mathrm{mem}}$ and $\sigma_{i}$ were estimated by parameter fitting.

## 3. Materials and Methods

### 3.1. Exosome Sample Preparation

Commercially available exosomes, bovine milk exosome (mean diameter: 119 nm, EXBM100L, COSMO Bio, Co., Ltd., Tokyo, Japan), human breast milk exosome (mean diameter: 174 nm, EXHM100L, COSMO Bio, Co., Ltd., Tokyo, Japan), and human breast cancer (MCF-7) exosome (peak diameter: 100 nm, EXOP-100A-1, System Biosciences, LLC, Palo Alto, CA, USA), were used. The exosomes were fluorescently labeled using ExoSparkler Exosome Membrane Labeling Kit-Green or Deep Red (Dojindo Laboratories, Kumamoto, Japan). Before the DEP experiments, the exosome suspensions were desalted using Amicon Ultra 0.5, 10 k (Merck KGaA, Darmstadt, Germany). The conductivity of the suspension medium was adjusted using NaCl solution.

### 3.2. DEP Experiments

DEP separation of human breast cancer exosomes from human breast milk exosomes was demonstrated. In the experiment, cancer and normal exosomes were labeled with deep red and green dyes, respectively. A mixture of cancer and normal exosomes was poured onto the microelectrode. The voltage was then applied at varying frequencies.

The crossover frequency was determined using a method similar to that used in our previous studies [5,27]. A Cr castellated microelectrode fabricated on a glass plate was used. The shortest gap between the microelectrodes was 5 µm. The microelectrode was placed on an epifluorescent microscope (IX71, Olympus, Co., Tokyo, Japan) equipped with a CCD camera (QIclick, QImaging, BC, Canada). A sinusoidal voltage, 20 V_PP_, was applied to the microelectrode from a function generator (WF1974, NF Corp., Kanagawa, Japan). The crossover frequencies of the exosomes suspended in various conductivity solutions were determined by direct observation. A total of 5 µL of the suspending solution was used for the observation. The frequency of the applied voltage varied from 100 kHz to 3 MHz with 100 kHz increments.

The determined $f_{\mathrm{xo}1}$ values were plotted against solution conductivity. Then, the measured values were parametrically fitted to Equation (5) using Kaleidagraph (Synergy Software, Reading, PA, USA) in which the fitting is carried out based on the Levenberg-Marquardt algorithm.

## 4. Results and Discussion

The DEP properties of bovine milk exosomes, human breast milk exosomes, and human breast cancer exosomes were investigated. As for the DEP property, the lower crossover frequency, $f_{\mathrm{xo}1},$ was determined by direct observation of the exosomes. To observe the exosomes, they were fluorescently labeled. After determining $f_{\mathrm{xo}1}$ in various conductivity solutions, the electrical parameters $C_{\mathrm{mem}}$ and $\sigma_{i}$ were estimated by parametric fitting to the theoretical curves.

To determine $f_{\mathrm{xo}1}$, we swept the applied frequency repeatedly. Because p- DEP was easily distinguished from the Brownian motion of the exosome, the frequency showing the p-DEP was determined first. Then, the applied frequency was decreased until the frequency caused no DEP motion. The determination was repeatedly carried out for the one measuring point. We compared the motion caused by p-DEP with Brownian motion to determine $f_{\mathrm{xo}1}$. This was due to the weakness of the n-DEP which caused errors in the measurements.

**Figure 2:**
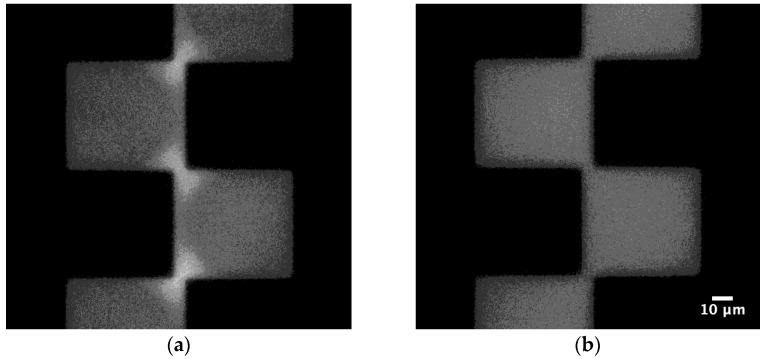
Typical images of exosome DEP. The fluorescent-labeled bovine milk exosomes were suspended in NaCl solution (20 µS/cm). (**a**) p-DEP at 1 MHz and (**b**) n-DEP at 100 kHz.

Figure 2 shows typical images of fluorescence-labeled exosomes from bovine milk under p- and n-DEP. When the voltage with a frequency causing p-DEP was applied, the exosomes moved to the shortest gap of the microelectrode where a strong electric field was formed. The bright spot in the gap decreased with decreasing frequency. When the applied frequency was lower than the crossover frequency, the exosomes were repulsed from the microelectrode and moved to the weaker electric field region. Exosomes exposed to n-DEP could not be observed clearly. The magnitude of the n-DEP force, which is the repulsive force from the microelectrode, caused difficulty in observation. As the magnitude of the electric field is inversely proportional to the distance, the magnitude of the DEP force decreased dramatically with an increase in the distance from the microelectrode. They were not condensed because the n-DEP force far from the microelectrode was weaker than that near the microelectrode.

The DEP of the exosomes was changed from n- to p-DEP by increasing the frequency around the crossover frequency. The DEP profile against the frequency showed that the measured frequency range corresponded to the range around the lower crossover frequency, $f_{\mathrm{xo}1}$. It was also confirmed that an exosome can be modeled as a single-shell model, not a homogeneous sphere model. Previous studies have revealed that a virus whose size is similar to an exosome can be considered in the homogeneous sphere model [5,7,8]. Previously surveyed viruses showed a single crossover frequency; the virus DEP was changed from p- to n-DEP by increasing the frequency around the crossover frequency. The virus DEP characteristics correspond to the homogeneous sphere model, similar to a polymer particle. In this case, the smaller the size of a homogeneous sphere, the more significant the influence of the surface conductance on its DEP profile is [28]. The measured DEP profiles revealed that the exosome can be modeled by the single-shell model. In this study, therefore, the observed exosome DEP characteristics were fitted to Equation (5) derived from the single-shell model.

**Figure 3:**
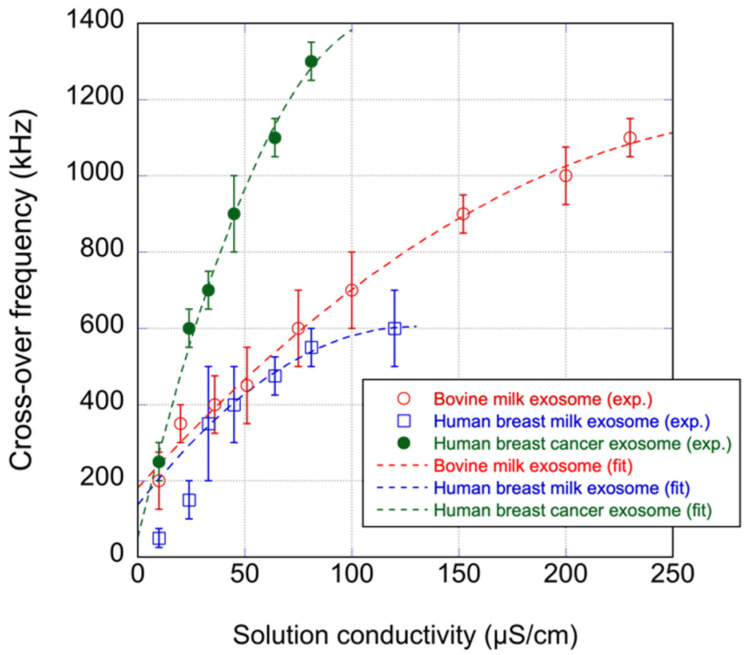
Plots of the crossover frequency against solution conductivity. Error bars indicate the standard deviation of the determined values of three independent experiments. The dotted lines are the best fitting curves to Equation (5).

A frequency ranging from 100 kHz to 3 MHz was applied with a 100 kHz increment to determine the crossover frequency. Exosomes were suspended in solutions of various conductivities. The conductivity was adjusted using NaCl. Figure 3 shows plots of the determined crossover frequency against the solution conductivity. A frequency higher than the crossover frequency caused p-DEP on the exosomes, whereas the lower frequency caused n-DEP. The bovine milk exosomes and human breast milk exosomes showed similar profiles. The human breast cancer exosome showed a profile different from the others.

The electrical parameters, $C_{\mathrm{mem}}$ and $\sigma_{i},$ were estimated by parametric fitting to the theoretical curves. The estimated values are listed in Table 1. $C_{\mathrm{mem}}$ and $\sigma_{i}$ of the bovine milk exosomes were almost twice those of the human breast milk exosomes, although the profiles of the crossover frequency were similar. The difference in the electric property could be due to the size of the exosomes. The diameters used to estimate were 119 nm and 174 nm for bovine and human breast milk exosomes, respectively.

For the reference, the $C_{\mathrm{mem}}$ and $\sigma_{i}$ of human breast cell (MCF-10A) and human breast cancer cell (MCF-7) are listed in Table 1 [29]. The estimated membrane capacitance, $C_{\mathrm{mem}}$, of the exosomes was almost identical to that of the host cells. This correspondence indicates that the exosome membrane is identical to that of the host cell. In addition, the electric property of the exosome membrane did not change at and after its release. This result suggests that the DEP investigation of exosomes can be used to diagnose cancer.

The conductivity of the inside exosome was much smaller than that of the host cell. Although the reason for this is unclear, it could be because of the concentration of the exosome components. It is well-known that many kinds of proteins, DNA, and RNA are present in exosomes. The volume ratio of those could influence the conductivity. There is another potential reason that some of the ions in the exosomes were released to the surrounding medium. The conductivity of the tested medium was adjusted to around 0.01 S/m (equal to 100 µS/cm). Human breast milk and breast cancer exosomes were provided as lyophilized forms. The process for lyophilization may vary the ion concentration inside the exosomes. In comparing the electric properties between the exosomes and their host cell, the membrane capacitance estimated from their DEP behaviors appears as values found in nature. This implies that the preparation process did not damage the exosome membrane, whereas it might vary with the components inside the exosome.

Figure 3 shows a significant difference in the crossover frequency between human breast milk and cancer exosomes. This suggests that when the frequency between $f_{\mathrm{xo}1}$ of cancer and the normal exosomes is applied, n-DEP occurs on the cancer exosomes, whereas p-DEP occurs on the normal exosomes. A mixture of cancer and normal exosomes was examined to confirm this. Human breast milk and cancer exosomes were stained with green and red fluorescent dyes, respectively.

**Figure 4:**
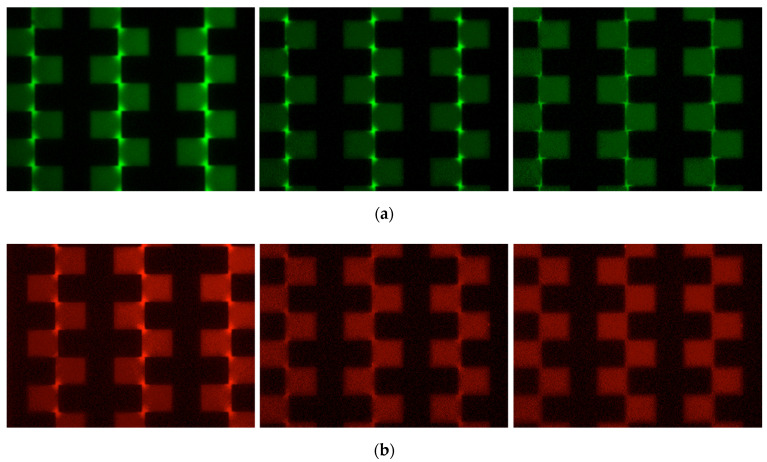
DEP separation of the exosomes. The human breast milk exosome (**a**, green) and the human breast cancer exosome (**b**, red) were mixed. The frequencies of the applied voltage were 1 MHz, 700 kHz, and 300 kHz (left to right). The photos are shown in pseudo-color images. The conductivity of the suspension medium was 40 µS/cm. The magnitude of the applied voltage was 20 V_PP_.

Figure 4 shows the images at various frequencies. In this experiment, the conductivity of the suspension medium was adjusted to 40 µS/cm. The filter cube in the microscope was changed to visualize each exosome. When the frequency of 1 MHz was applied, both exosomes showed p-DEP. In the case of the cancer exosomes, the bright spots in the electrode gap dramatically decreased with decreasing frequency. At 300 kHz, the bright spots of the cancer exosomes disappeared, whereas the bright spots of the normal exosomes were distinguished.

The separation result implies practical DEP-based separation of cancer exosomes. In the system, normal cells suspended in the sample solution will be removed by centrifugation first. Then, the supernatant, including exosomes, will be applied to a two-step DEP separation. The normal exosomes will be trapped on the first electrode by p-DEP at a low frequency (300 kHz). The cancer exosomes will then be recovered using DEP at a high frequency (1 MHz) at the second electrode. Because the separation is based on the electrical properties of cancer exosomes, it identifies the exosomes from cancer cells. The capture of cancer exosomes could be monitored as the impedance change of the microelectrode, such as the DEP-based virus measurement [5].

In this study, the crossover frequencies of the exosomes were determined by direct observation. To visualize the exosomes, they were stained with fluorescent dyes attached to the exosome membrane. This can influence the electrical properties of the membrane. As a result, the estimated membrane capacitances were very close to those of the host cells. Moreover, a previous study revealed that the zeta potential of the membrane was not influenced by the dye [31]. We considered that the dye did not significantly influence the DEP properties of exosomes.

In this study, the size of the fluorescently labeled exosomes was not determined. The labeling process and ion concentration may cause the aggregation of the exosomes. If aggregation does occur, it does not influence Re[K] in theory. Therefore, confirmation of the aggregation is not necessary for this study. The typical diameters provided by the instruction manuals were used to estimate the electric property using Equation (5). However, the exosome size was distributed between 50 to 200 nm and the distribution can influence the accuracy of the estimation. This point must be addressed in future work.

## 5. Conclusions

This study investigated the crossover frequencies of the following three types of exosomes: bovine milk exosomes, human breast milk exosomes, and human breast cancer exosomes. As a result, bovine and human milk exosomes showed similar DEP properties, whereas exosomes from cancer cells were significantly different from the others. Using the experimentally determined crossover frequency, the membrane capacitance and conductivity of the exosomes were estimated. The estimated value revealed a significant difference in the electrical properties of bovine and human milk exosomes, even though the DEP properties were similar. It was found that the membrane capacitances of human breast milk and cancer exosomes were almost identical to those of their host cells. The conductivity of the exosome was much lower than that of the host cell. Based on these results, the DEP separation of exosomes was demonstrated. The results demonstrated that DEP can be utilized to determine and separate the exosomes released from cancer cells for liquid biopsy. Additionally, our method can be utilized to estimate the electric property of other types of extracellular vesicles if their size and DEP property are determined

## Figures and Tables

**Table 1:** Membrane capacitance, $C_{\mathrm{mem}}$, and inner conductivity, $\sigma_{i}$, of exosome samples.

Types	$C_{\mathrm{mem}}\;[\mathrm{mF}/m^{2}]$	$\sigma_{i}\;[S/m]$	Reference
Bovine milk exosome	61.2	0.0315	This work
Human breast milk exosome	35.9	0.0130	This work
Human breast cancer exosome	19.4	0.0117	This work
Human breast cell (MCF-10A)	39.4	1.4 *	[29]
Human breast cancer cell (MCF-7)	19.5	1.3 *	[29]
	12.4	0.23 *	[30]

* Conductivity of the cytoplasm.

## Data Availability

The data that support the findings of this study are available from the corresponding author (M.N.).
